# A visual scale to rate amygdalar atrophy on MRI

**DOI:** 10.1007/s00330-024-11249-7

**Published:** 2024-12-19

**Authors:** Francesca B. Pizzini, Federica Ribaldi, Valerio Natale, Max Scheffler, Vittoria Rossi, Giovanni B. Frisoni

**Affiliations:** 1 Radiology and Department of Engineering for Innovation Medicine, Verona University, Verona, Italy; 2 Geneva Memory Center, Department of Rehabilitation and Geriatrics, Geneva University Hospitals, Geneva, Switzerland; 3 Laboratory of Neuroimaging of Aging (LANVIE), University of Geneva, Geneva, Switzerland; 4 Department of Diagnostic and Public Health, Rivoli Hospital, Rivoli (TO), Italy; 5 Division of Radiology, Geneva University Hospitals, Geneva, Switzerland

**Keywords:** Amygdala, Dementia, MRI, Atrophy

## Abstract

**Background:**

Visual rating scales are routinely used in clinical radiology to assess brain atrophy on scans of patients with suspected neurodegenerative conditions. Limbic predominant age-related TDP-43 encephalopathy (LATE) has recently been described, featuring early and severe atrophy of the amygdala. However, there is currently no scoring system specifically designed to assess amygdalar atrophy on MRI.

**Objectives:**

to develop and validate a visual rating scale for amygdalar atrophy.

**Materials and methods:**

Stringent criteria were developed for no, mild/moderate, and severe amygdalar atrophy based on axial and coronal volumetric T1-weighted MRI scans. Inter- and intra-rater reliabilities were estimated by three independent expert neuroradiologists in 100 randomly selected scans from the Geneva Memory Center cohort selected to be representative of the variability of medial temporal atrophy. Convergent validity was evaluated versus amygdalar volumes extracted by FreeSurfer on 1943 consecutive patients. Criterion validity versus autopsy-confirmed LATE neuropathologic changes were studied in the pathological subset of the Alzheimer’s Disease Neuroimaging Initiative (ADNI) cohort (*N* = 96).

**Results:**

Intra- and inter-rater agreements of amygdalar visual ratings were between substantial and almost perfect (weighted Cohen’s Kappa 0.71 to 0.93). Visual ratings were strongly associated with amygdalar volumes (*p* ≤ 0.001 on the Kruskal–Wallis test). LATE neuropathologic changes were associated with visual ratings of amygdalar atrophy (*p* = 0.057 on a test for trend).

**Conclusion:**

The proposed visual amygdalar atrophy scale is a reliable and valid tool to assess amygdalar atrophy on MRI and can be a useful adjunct in routine radiological reporting.

**Key Points:**

***Question*** *Assessment of amygdalar atrophy is crucial for diagnosing neurodegenerative diseases, as the limbic predominant age-related TDP-43 encephalopathy, yet no validated visual rating scale exists.*

***Findings*** *The proposed amygdalar atrophy scale demonstrated high intra-rater and inter-rater reliability, strong correlation with amygdalar volumetry, and association with limbic predominant age-related TDP-43 encephalopathy (LATE).*

***Clinical relevance*** *The amygdalar atrophy scale provides a reliable practical assessment tool that enhances diagnostic accuracy for dementia-related conditions, particularly aiding in identifying limbic predominant age-related TDP-43 encephalopathy.*

## Background

Visual rating scales are an essential part of the diagnostic assessment of patients with suspected cognitive disorders [1–8]. National and international guidelines and task forces [2, 6, 9, 10] have proposed visual scales for evaluating medial temporal lobe atrophy, and their introduction into clinical-radiological practice has strengthened the role of magnetic resonance imaging (MRI) in the diagnostic workup process of patients with neurodegenerative disorders.

Recently, a relatively new condition, called limbic age-related transactive response DNA binding protein of 43 kDa (TDP-43) encephalopathy (LATE), has been described. This is thought to be a proteinopathy characterized by the deposition of TDP-43 in the medial temporal lobe that subsequently extends to the temporal cortex and beyond [11–13]. The earliest site of TDP-43 deposition is the amygdala, and the macroscopic feature consists of severe amygdalar atrophy. It is important for us radiologists to specifically assess amygdalar atrophy. Even if the amygdala is one of the temporo-mesial structures, atrophy assessment using the Scheltens scale is based on the assessment of three other parameters: width of the choroid fissure and of the temporal horn of the lateral ventricle and the height of the hippocampus [14]. Thus, if one of the three features on which the scale is based is the hippocampus itself, the amygdala is not specifically considered.

The objectives of this study are to develop a visual rating scale for amygdalar atrophy (amygdalar atrophy scale, AAS) that can be easily used in clinical practice and provide evidence of analytical validity. To this end, we used brain MR scans from a large cohort of memory clinic patients to evaluate (i) its inter- and intra-rater reliability, (ii) its convergent validity versus volumetry, and (iii) its criterion validity.

## Methods

### Study design and participants

This is a retrospective validation study in the cohort of patients of the Geneva University Hospital Memory Center (GMC) who provided voluntary written informed consent to be included in the study [15]. The study was approved by the local ethics committee (PB_2016-01346, 2020-00403) and conducted in accordance with the principles of the Declaration of Helsinki and the International Conference on Harmonization Good Clinical Practice.

In this cohort we assessed the inter/intra-rater reliability of the AAS and its convergent validity versus automated volumetry. Criterion validity was evaluated versus autopsy-confirmed LATE neuropathologic changes (LATE-NC) in the autopsy-confirmed Alzheimer’s Disease Neuroimaging Initiative (ADNI) cohort.

#### Geneva Memory Center cohort

**Fig. 1:**
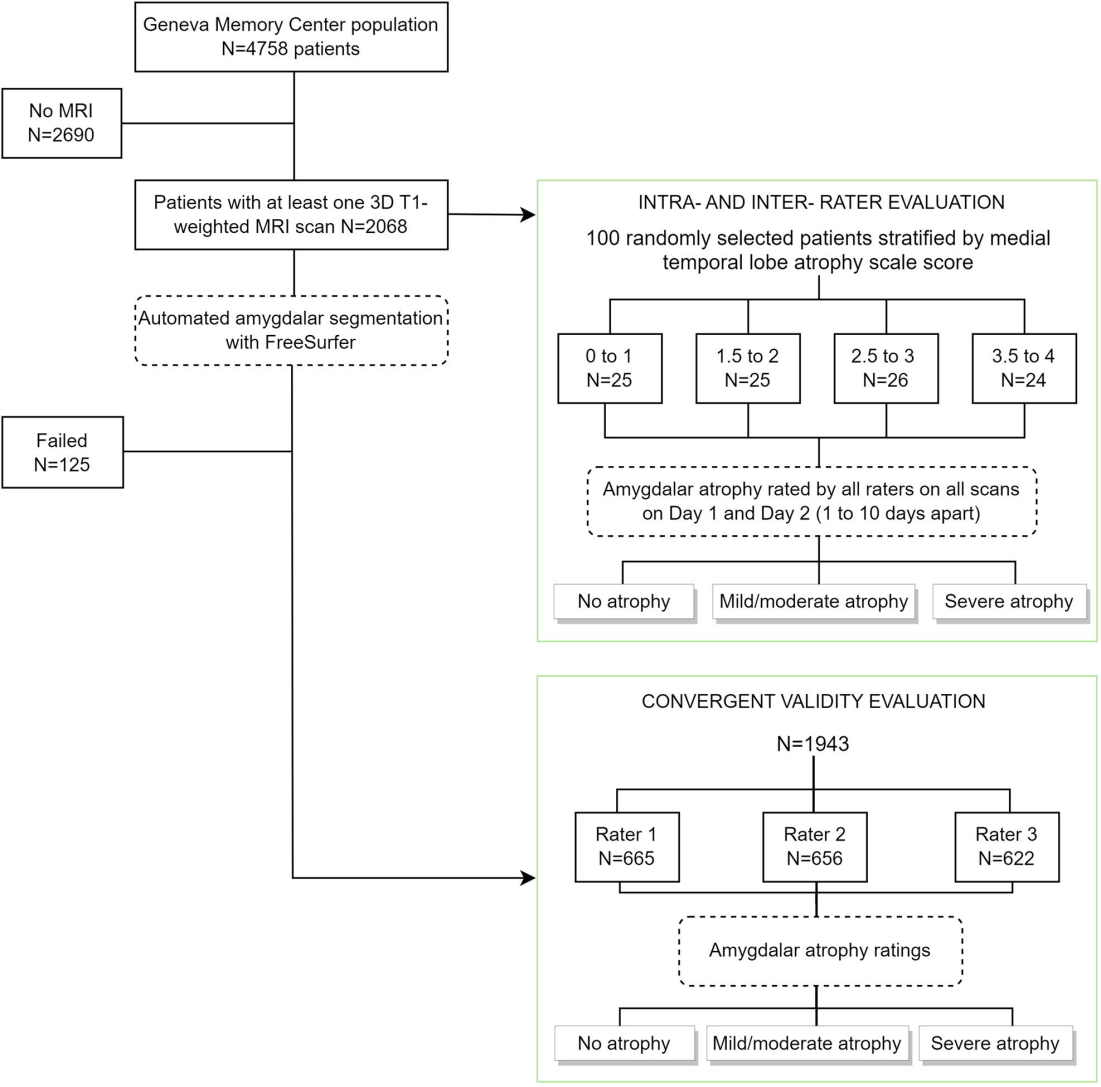
Design and scan selection for the study of intra and inter-rater agreement and convergent validity

The GMC cohort consists of individuals consulting the Geneva Memory Center for cognitive concerns along the continuum from no cognitive impairment to mild cognitive impairment (MCI), and dementia, as reported in Fig. 1. All underwent in-depth neurological, clinical, and neuropsychological evaluation as described elsewhere [15].

Of the 4758 individuals that have been consulted at the GMC between 2014 and 2022, 2068 had available MRI scans.

MRIs were acquired on a 3-T MRI scanner (Magnetom Skyra, Siemens Healthcare) with a 64-channel head coil at the Division of Radiology at Geneva University Hospitals. Acquisition parameters of the T1-weighted 3D sequence were TR/TE = 1930/2.36 ms; flip angle = 8°; resolution = 0.9 mm isotropic; 208 sagittal slices.

Of the abovementioned 2068 exams, 100 were randomly selected to evaluate the inter- and intra-rater reliability of the AAS. To achieve a comprehensive representation of the severity of amygdalar atrophy, we randomly selected an equal number of scans per each of four severity levels (0–1, 1.5–2, 2.5–3, and 3.5–4) on the medial temporal atrophy (MTA) scale scored by blinded expert neuroradiologists in the context of routine care. These scans were randomly presented to the amygdalar atrophy raters with respect to the MTA severity.

Convergent validity was tested against amygdalar volumes computed using FreeSurfer (v 7.1.0 http://surfer.nmr.mgh.harvard.edu/ [16]). This analysis included all individuals with available MRI scans. FreeSurfer has proven to be a reliable tool for amygdala segmentation, although it overestimates amygdala volumes by 61% compared to manual segmentation [17]. The volumes of the left and right amygdala were adjusted by the participant’s total intracranial volume to control for differences in head size and calculated separately and the most affected side compared with the visual scores. Automated segmentation failed for 125 cohort participants, resulting in 1943 valid cases for convergent validity analysis.

#### ADNI cohort

Participants included in the neuropathological core cohort of ADNI (adni.loni.usc.edu, http://adni.loni.usc.edu/about/#core-container [18]) were used to evaluate criterion validity. The ADNI was launched in 2003 as a public-private partnership, led by Principal Investigator Michael W. Weiner, MD. The primary goal of ADNI was to test whether serial magnetic resonance imaging (MRI), positron emission tomography (PET), other biological markers, and clinical and neuropsychological assessment can be combined to measure the progression of Alzheimer’s disease (AD).

Ninety-six neuropathological core ADNI participants had a neuropathological examination of TDP43 and a T1 MRI scan available by June 2023. For the aim of the current study, the presence of TDP-43 pathology was defined by the presence of TDP-43-immunoreactive inclusions in any of the following regions, known to be sites of TDP-43 pathology in LATE: amygdala, hippocampus, entorhinal cortex/inferior temporal gyrus, and neocortex. A composite measure was computed as the sum of the presence of TDP-43 pathology in each region, resulting in a global score that ranged from 0 (absence of TDP-43 pathology in all regions) to 4 (presence of TDP-43 pathology in all regions). LATE-NC was defined as present if TDP-43 inclusions were found in the following regions: amygdala, hippocampus, or entorhinal/inferior temporal cortex, neocortex [19].

When more than one T1-weighted MRI scan was available, the one closest to death was selected. On average, the time gap between the MRI scan and neuropathologic assessment was approximately 3 years.

### Visual rating scale

ITK-SNAP 3.X was used for T1-weighted MRI scan visualization.

The normal amygdala is an almond-shaped structure in the anterior part of the mesial temporal lobe, adjacent to the lateral ventricle’s temporal horn [20, 21], that appears irregularly oval in the axial sections and roughly rounded or oval on the coronal sections, passing through the center of this structure. Amygdalar atrophy is manifested by a loss of normal volume that, in its most severe degree, reaches a lamellar shape in both sections. Any volume loss is accompanied by enlargement of the adjacent temporal horn of the lateral ventricle.

**Fig. 2:**
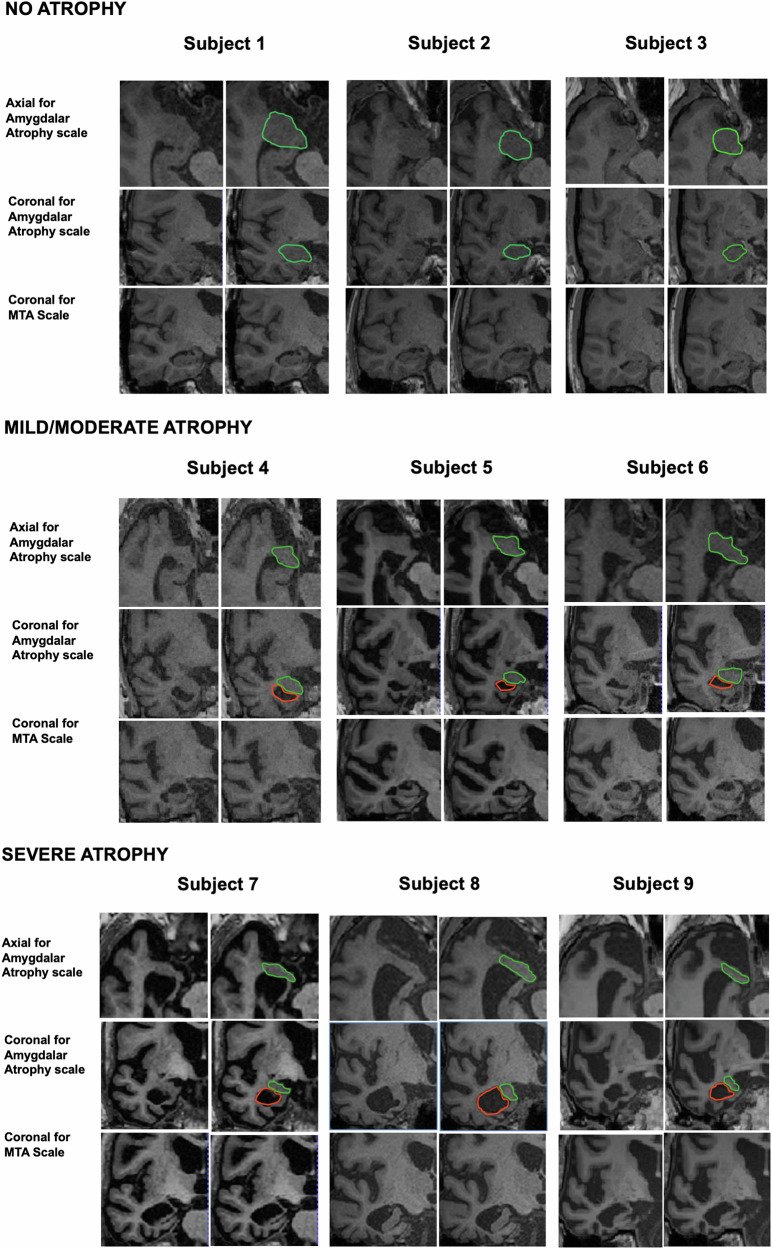
Some examples of images highlighting the characteristics of the different ranges of normal and abnormal amygdala morphology used in the classification system (subjects 1 to 3, no atrophy; 4 to 6, mild-moderate; and 7 to 9, severe). For each subject in the right column, amygdala morphology is underlined in green, and any presence of the comma sign is in orange

**Fig. 3:**
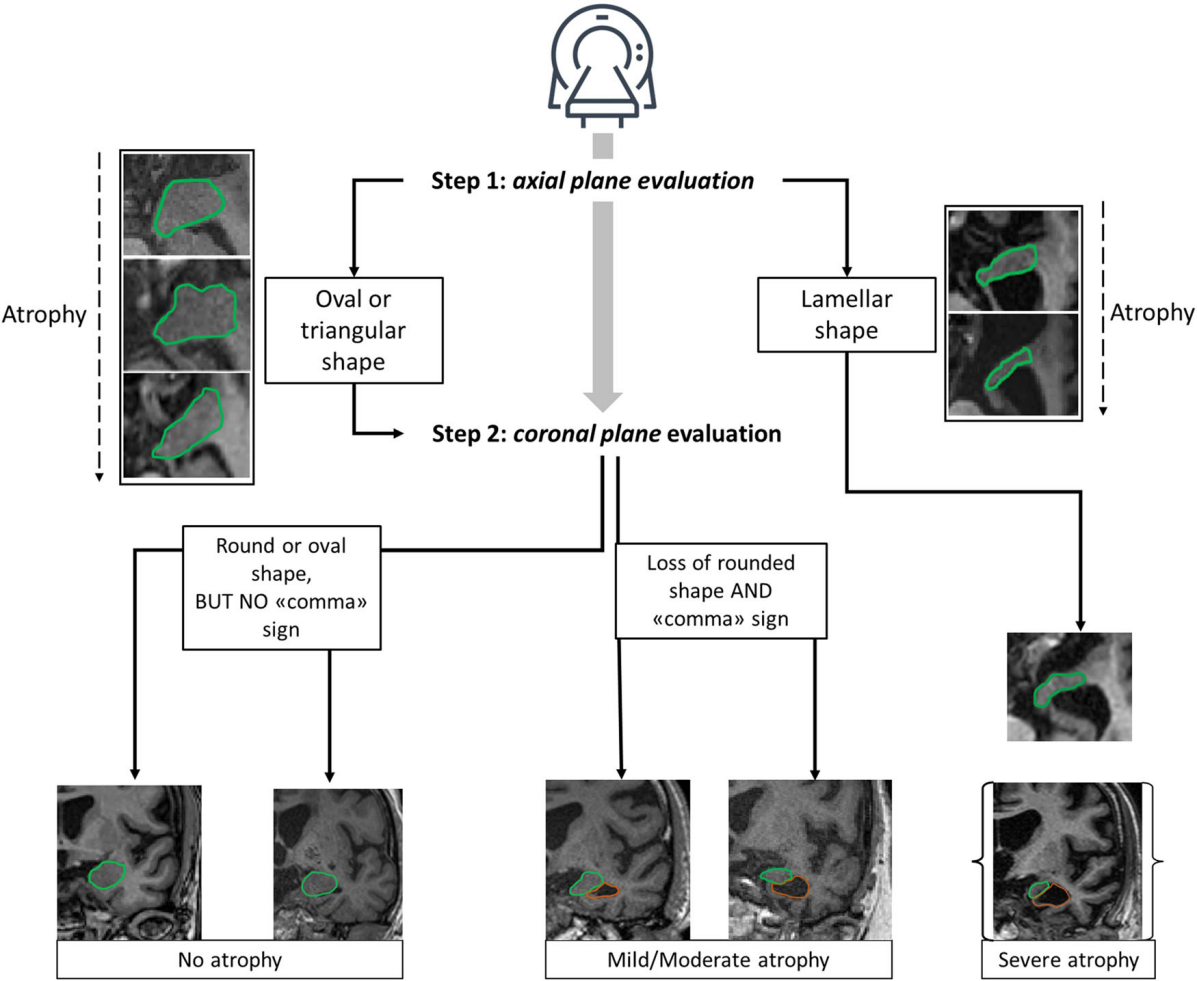
Rating procedure for the amygdalar atrophy scale (AAS). Rating starts with the search for severe atrophy of the amygdala on an axial plane cutting through the dorsoventral midpoint of the amygdala (Step 1). If no severe atrophy is detected, Step 2 involves distinguishing normal size and shape from mild to moderate atrophy on a coronal plane cutting through the rostrocaudal midpoint of the amygdala. The atrophy score is based on the most atrophic side. The “comma sign” indicates an enlargement of the tip of the temporal horn, taking on a comma-like shape on coronal scans

In our work, amygdalar atrophy was assessed on a 3-level scale (no atrophy; mild/moderate atrophy; severe atrophy—Fig. 2) following a standardized approach as described in the flowchart (see Fig. 3). Visual evaluation was performed in the axial and coronal planes because they allow simultaneous delineation of the morphology of the amygdalae, the adjacent portion of the temporal horns, and the CSF cisternal spaces, all of which are essential for assessing progressive degrees of atrophy.

The first step in the scoring process was to recognize each amygdala in the axial plane, to select the plane that cuts the dorsoventral midpoint of the amygdala, and to identify its shape. A score of *severe atrophy* was assigned if the normal ovalar ‘almond’ shape had been lost and replaced by a ‘lamellar shape’. In all other cases (which generally showed a roughly oval or triangular shape), a second step was necessary to distinguish between absent and mild/moderate atrophy.

This second phase involved then the use of a coronal plane that cuts the rostrocaudal midpoint of the amygdala and the assessment of its morphology rounded in this plane: a score of *no atrophy* corresponded to a roughly rounded shape of the amygdala OR an oval morphology but without enlargement of the adjacent temporal horn. A *mild/moderate atrophy* score corresponded to a loss of the rounded shape of the amygdala with enlargement of the adjacent temporal horn (“comma sign”). The most atrophic side, right or left, was considered for the final score.

The intra-rater reliability of the AAS was evaluated by three raters (R1, R2, and R3). These were neuroradiologists with 20, 10 years, and 1 year of reporting experience, respectively. Before the reliability scoring on the 100 randomly selected images, the raters held a training session on 30 cases to agree on the evaluation approach and discuss discordant scans. These 30 cases were excluded from the other analyses. The same raters were then asked to rate the 100 randomly selected scans twice on two different days, at least 1 day apart. To prevent rater bias, raters were blinded to the clinical characteristics of the patients and their own previous ratings.

Furthermore, the convergent validity of the AAS was assessed by the same R1, R2, and R3, who blindly evaluated 665, 656, and 662 images, respectively. In the criterion validity study of the ADNI pathological cohort, only R1 evaluated the 96 scans.

Figure 1 illustrates the study’s design and scan selection for the assessment of intra- and inter-rater agreements and convergent validity.

### Statistical analysis

Pair-wise intra- and inter-rater agreements were estimated with the quadratic weighted Cohen’s Kappa (wK). Fleiss’s Kappa coefficient was computed to evaluate inter-rater agreement among all three raters. Cohen’s Kappa values ≤ 0 indicated no agreement, 0.01–0.20 none to slight, 0.21–0.40 fair, 0.41–0.60 moderate, 0.61–0.80 substantial, and 0.81–1.00 almost perfect to perfect agreement.

The convergent validity metric was the difference of mean volumes of the amygdala by AAS severity score. Statistical significance was assessed with Kruskal–Wallis test and post-hoc pairwise comparisons by Dunn’s test. The non-redundancy of the AAS and MTA ratings was investigated using a two-way ANOVA to test the statistical differences in amygdala volume among the three levels of AAS (0, 1, 2) and the three levels of MTA (0–2, 3, 4), including the interaction between AAS and MTA. Post-hoc analysis to verify the differences between pairs was performed using Kruskal–Wallis test and pairwise comparisons by Dunn’s test.

Criterion validity was evaluated as the association of the prevalence of LATE-NC with AAS severity scores with a test for trend for proportions.

Statistical analyses were carried out with R version 4.3.1.

## Results

The final GMC cohort after the application of the inclusion/exclusion criteria is described in Fig. 1 and participants’ demographics, clinical, and imaging characteristics of the GMC and ADNI cohorts are summarized in Table 1. Participants of the former had demographic, clinical, and imaging features in line with what is expected for a typical memory clinic population. Interestingly, the mean mini-mental state examination (MMSE) score for this group was 25/30, and almost one-quarter had no cognitive impairment, consistently with the trend towards increasingly earlier referral of patients with cognitive concerns in the past few decades [22–24].

**Table 1:** Demographics, clinical, and imaging features of the study cohorts

Variables		Geneva Memory Center cohort (*N* = 1943)	ADNI neuropathological core cohort (*N* = 96)
Age, years	At MRI	71 ± 11	80 ± 7
	At neuropathology	-	83 ± 7
Gender, female—*n* (%)		1136 (59%)	23 (24%)
Education, years		13 ± 4	16 ± 3
Diagnosis, *n* (%)			
Cognitively unimpaired		458 (23%)	14 (15%)
Mild cognitive impairment		777 (40%)	49 (51%)
Dementia		401 (21%)	33 (34%)
Unknown		307 (16%)	-
Mini-mental state examination		25 ± 5	21 ± 7
Medial temporal lobe atrophy scale		1.2 ± 1.0	-
Amygdalar volume (mm^3^)		1250 ± 291	-

Values denote mean ± standard deviation or number (percentage)

*MRI* magnetic resonance imaging

As expected, the ADNI pathological cohort was more than 10 years older at death, and the MMSE was lower than that of the living cohort. The proportion of cognitively unimpaired, MCI and dementia reflected the selection criteria of the ADNI cohort (1:2:1), skewed by cognitive progression towards the cognitively more severely impaired groups.

**Fig. 4:**
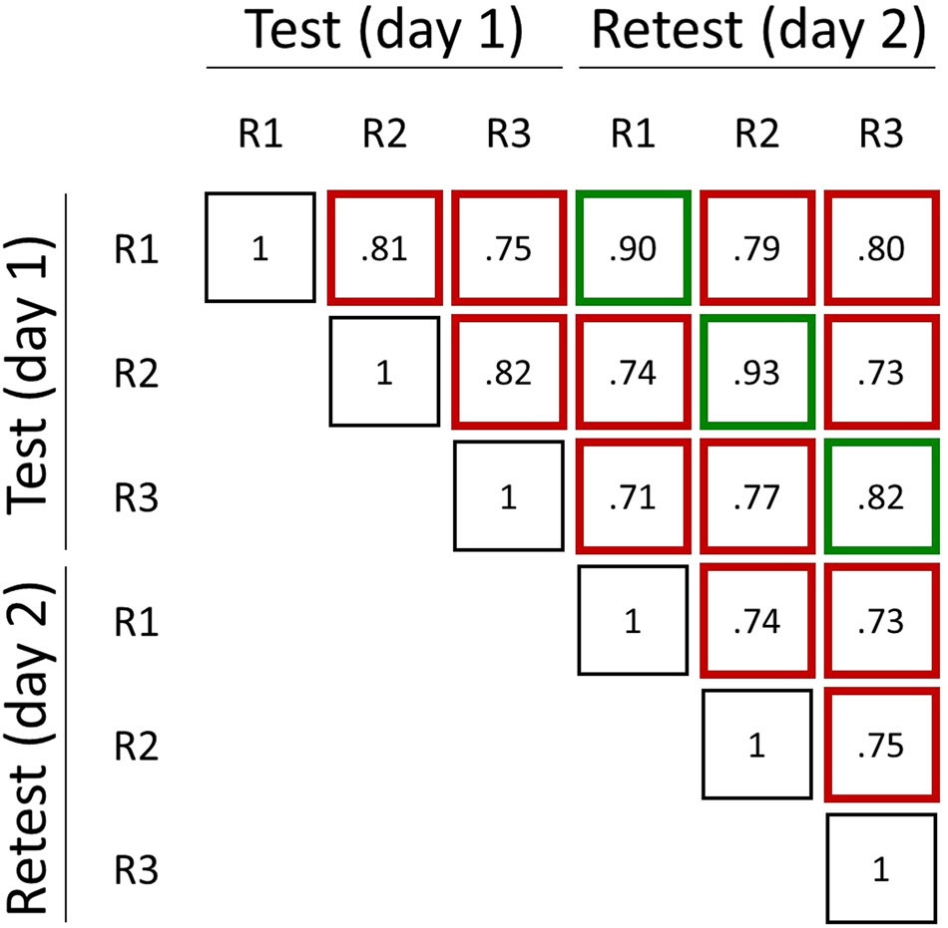
Rater agreement matrix. Test-retest on the diagonal (green) denotes intra-rater agreements. All other cells (red) denote inter-rater agreements. Values denote weighted Cohen’s kappa. R1, R2, R3: Rater 1, 2, 3

The intra-rater agreement analysis (Fig. 4) shows a high level of agreement (almost perfect), with wK values of 0.90, 0.93, and 0.82 for raters 1, 2, and 3, respectively. Pair-wise inter-rater agreements were *substantial* both on test (wK between 0.75 and 0.82) and retest (wK between 0.73 and 0.75). The absolute agreement on the test ratings was 73%, with a Fleiss’ Kappa of 0.70, indicating substantial agreement. The absolute agreement on the retest scores was 69% with a Fleiss’ Kappa of 0.64, again denoting substantial agreement.

**Fig. 5:**
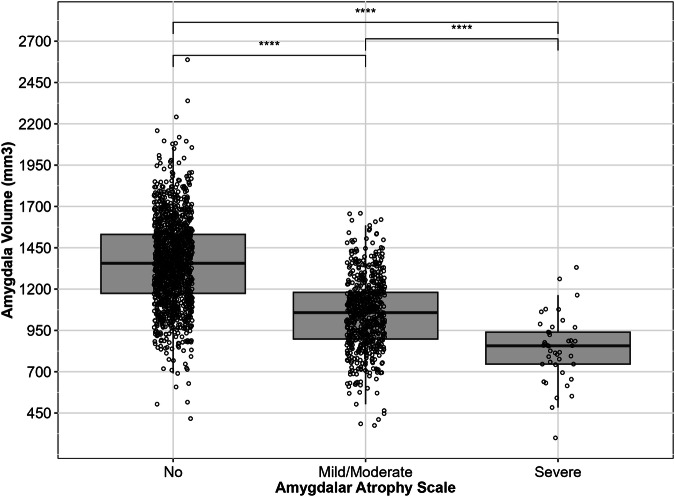
Convergent validity of the amygdalar atrophy scale versus amygdalar volumes. All group comparisons are highly significant on the Kruskal–Wallis test (*p* < 0.0001). SD, standard deviation

Figure 5 shows decreasing amygdalar volumes of the most affected side among individuals without atrophy, with mild/moderate atrophy, and severe atrophy. All pair-wise group comparisons were statistically highly significant. Supplementary Fig. 1 shows that the results hold true even after disaggregating by rater (*p* ≤ 0.05). The non-redundancy of AAS and MTA ratings is shown in Supplementary Table 1 and Supplementary Figs. 2 and 3. Statistically significant differences in amygdala volumes were observed across both AAS and MTA categories (*p* < 0.001), and the interaction between AAS and MTA was also significant (*p* = 0.039). Post-hoc comparisons showed significant differences in amygdala volumes among AAS scores within each MTA group, except between mild/moderate amygdalar atrophy and severe amygdalar atrophy within the MTA 0-1-2 group (*p* = 0.252), and between no atrophy and mild/moderate atrophy in the MTA 3 (*p* = 0.060) and MTA 4 (*p* = 0.300) groups. Moreover, Supplementary Table 1 and Supplementary Fig. 2 show that an MTA score of 4 does not predict severe amygdalar atrophy, with as many as 56% of patients with MTA = 4 having shown none to moderate amygdalar atrophy. Conversely, while the vast majority of scans with an MTA score of 3 had moderate to no amygdalar atrophy, a non-negligible 8% of scans had severe amygdalar atrophy. Severe amygdala atrophy was found to be exceedingly rare in scans with MTA of 2 or lower.

**Fig. 6:**
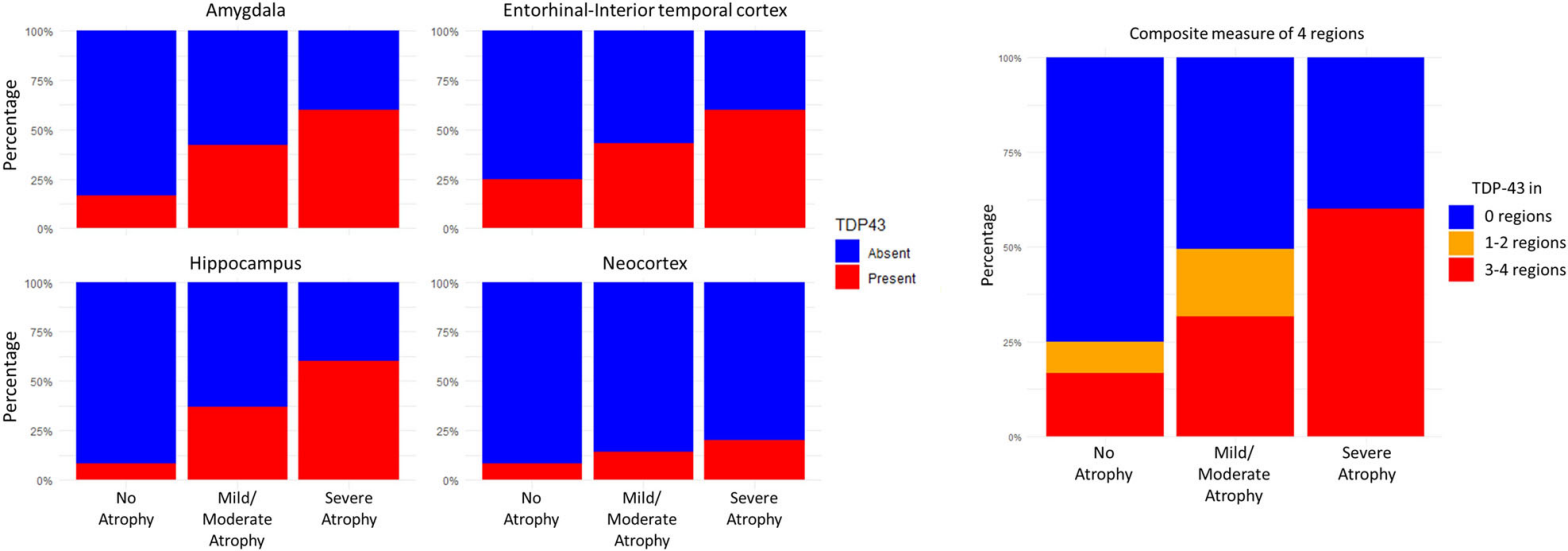
Association of amygdalar atrophy scale ratings on MRI with TDP-43 pathology in the 96 brains of the ADNI neuropathology core cohort. Bar plots represent the percentage of individuals with evidence of TDP-43 pathology in brain regions selected for relevance to LATE (limbic-predominant age-related TDP-43 encephalopathy). Scans showing no, mild/moderate, and severe atrophy were 12, 79, and 5

In the ADNI cohort, out of 96 subjects, 45 presented LATE-NC. A linear association was found between TDP-43 pathology with increasing amygdalar atrophy in the neuropathology core ADNI cohort (Fig. 6). The association was statistically significant in the hippocampus (*p* = 0.022) and close to the 0.05 threshold for statistical significance in the amygdala (*p* = 0.057). The linear associations in the entorhinal/inferior temporal cortex, neocortex, and composite were above the 0.05 threshold, likely for the small number of scans with severe amygdalar atrophy.

## Discussion

We developed and validated in our study a new rating scale for amygdalar atrophy, assessed in two planes, the axial and coronal, that can be easily included in routine radiological practice. Although many FDA and CE-approved software tools for clinical use offer separate volumetric measurements for the hippocampus and amygdala, there are currently no specific radiological visual scales for amygdalar atrophy. In particular, the widely used MTA scale does not take amygdalar atrophy into account as it is assessed in only one plane, the coronal. This plane is positioned parallel to the brainstem axis at the level of the anterior pons and includes the hippocampus, but not the amygdala [14]. In fact, the association of the Scheltens scale (on 5 levels from 0 to 4) with the AAS (on 3 levels from 0—no atrophy—to 2—severe atrophy), showed that only 44% of cases with an MTA score of 4 and 8% of cases with an MTA score of 3 also had the highest AAS score of 2 (severe atrophy). It follows that in the radiological assessment of MTA, the use of the Scheltens scale does not make the AAS redundant, and the use of both is indicated.

A visual scale specific to the amygdala is crucial in the radiological assessment of neurological and neuropsychiatric disorders, particularly in conditions where this structure plays a key role, as seen in LATE. This is a relatively frequent proteinopathy characterized by TDP-43 deposits in the amygdala, CA1 of the hippocampus, subiculum, entorhinal cortex, and other extralimbic structures, accompanied by amygdalar atrophy with or without hippocampal sclerosis [11]. In LATE, amygdalar atrophy is often asymmetric, and medial temporal lobe atrophy shows a rostrocaudal gradient, as reported in a number of neuropathological studies. The shape of the amygdala typically appears oval in the axial plane, whereas, in a coronal plane cutting through its dorsoventral midpoint, it is rounded. With the onset of atrophy, the morphology of the amygdala progressively changes in both planes, until it reaches a lamellar appearance in the most severe of cases.

The visual scale developed in this study showed high repeatability both within and between raters, a crucial characteristic for the adoption of a rating instrument for routine radiological assessments. Interestingly, the agreement coefficients of this AAS are compared favorably with those of other visual rating scales that are widely utilized in routine radiological assessments. Scheltens et al [14] assessed inter-rater agreement between four raters on the visual assessment of MTA on Tl-weighted coronal MRI scans of 100 MRI studies with a 4-point scale and reported an inter-observer concordance expressed by *K*-values of 0.44 (95% CI = 0.34–0.54) and 0.51 (95% CI = 0.41–0.61). In Kapeller et al [1], three raters with different levels of experience tested the interrater agreement of three established rating scales on age-related white matter changes (ARWMC) of 74 subjects at baseline and found an interrater agreement for all scales, with *K*-values ranging from 0.59 to 0.78. Koedam et al [3] reported the high intra-rater agreement (between 0.93 and 0.95) and inter-rater concordance (between 0.65 and 0.84) between three raters in the assessment of posterior atrophy, medial temporal lobe atrophy (MTA) and global cortical atrophy using MRI scans of 118 memory clinic patients.

Importantly, despite one of the three raters being relatively inexperienced, both intra- and inter-rater agreement coefficients in our study fell within the substantial range or exceeded it.

The AAS demonstrated a strong correlation with the volume of the amygdala, measured by Freesurfer [17], although in this paper, Alexander et al noted that this software may overestimate amygdala volume compared to manual measurements. However, in our results, this bias is more pronounced for smaller amygdalae than for larger amygdalae, which, if anything, may have attenuated the association of volumes we found with visual assessment scores.

It should be emphasized that a strong correlation with volumetry is a feature of other visual rating scales widely used in the radiological routine. Kapeller et al [1] evaluated the correlation between visual assessments and quantitative volumetric ARWMC measurements of 255 MRI scans and demonstrated significant agreement between visual assessment and quantitative volumetric ARWMC measurement (Kendall *W*, 0.37, 0.48 and 0.57; *p* = 0.001). A recent systematic review and meta-analysis reported that no significant difference was observed between the MTA scale and hippocampal volumetry (automatically or manually calculated) with regard to their sensitivity and specificity in differentiating AD from healthy controls (sensitivity: 73% of MTA vs. 81% of volumetry; specificity, respectively 90% vs. 85%) (*p* = 0.40) [25].

A strong point of this study is the validation of the AAS versus pathology. While the group size of the neuropathology core ADNI cohort is relatively small, a clear linear association was found between visually rated amygdalar atrophy and the presence of TDP-43 pathology in key brain regions. Not all visual rating scales currently used in radiological reporting of MRI scans of cognitive patients have this degree of evidence. In fact, to our knowledge, anatomopathological correlations with visual scales are present in few studies. In Barkhof et al [26], the MTA scale was performed on 132 brains fixed in formalin and subjected to post-mortem magnetic resonance imaging, and a strong relationship was found between MTA scores and Alzheimer’s pathology (*p* < 0.001), proving sensitive to primary degenerative pathology of the hippocampus, but not specific to Alzheimer’s type pathology. These results are consistent with other previous studies correlating imaging with post-mortem hippocampal volume [27, 28].

A limitation of our study includes the lack of correlation with clinical data that can test the scale’s diagnostic utility in distinguishing LATE from patients with other forms of cognitive impairment. Indeed, the lack of in-vivo biomarkers of TDP-43 pathology is what led us to use a pathologic cohort to show the criterion validity of the AAS. PET tracers for TDP-43 are under development and, when available on a large scale, will allow in-vivo demonstration of validity. Another limitation is that the diagnostic relevance of mild/moderate amygdala atrophy remains unclear. However, we have demonstrated that severe atrophy on the AAS conveys non-redundant information versus the MTA scale, at least in brains with an MTA of 3 and higher. A clinical use of the AAS could thus be hypothesized where the MTA scale is used first, and the AAS follows in cases of MTA of 3 or higher. More work is warranted to disentangle the mild from the moderate stage of amygdalar atrophy and understand their diagnostic relevance. Last, the generalizability of the AAS to non-memory clinic populations (e.g. community-dwelling persons, psychiatric patients, patients with traumatic brain injury, epilepsy, etc.) will need to be demonstrated.

In conclusion, the amygdalar atrophy scale is a valid measure of the volumetric reduction of the amygdala that can be used in the clinical MRI reporting of memory clinic patients.

## Supplementary information

ELECTRONIC SUPPLEMENTARY MATERIAL

## Abbreviations

AAS: Amygdala Atrophy Scale
AD: Alzheimer’s disease
ADNI: Alzheimer’s Disease Neuroimaging Initiative
ARWMC: Age-related white matter changes
LATE: Limbic age-related TDP-43 encephalopathy
MCI: Mild cognitive impairment
MMSE: Mini-mental state examination
MTA: Medial temporal atrophy
TDP-43: Transactive response DNA binding protein of 43 kDa
