# Supplementary material for: A visual scale to rate amygdalar atrophy on MRI
Source: Eur Radiol. 2024 Dec 19;35(7):4246–56. doi: 10.1007/s00330-024-11249-7 (PMC12165972; doi:10.1007/s00330-024-11249-7)
Supplement: Supplementary file 1 — ELECTRONIC SUPPLEMENTARY MATERIAL [file 330_2024_11249_MOESM1_ESM.pdf]

A visual scale to rate amygdalar atrophy on MRI

Electronic Supplementary Material

Supplementary Figure 1. Convergent validity of the amygdalar atrophy scale (AAS) versus amygdalar volumes by rater. All group comparisons were highly significant on Kruskal-Wallis test ($p<0.005$).

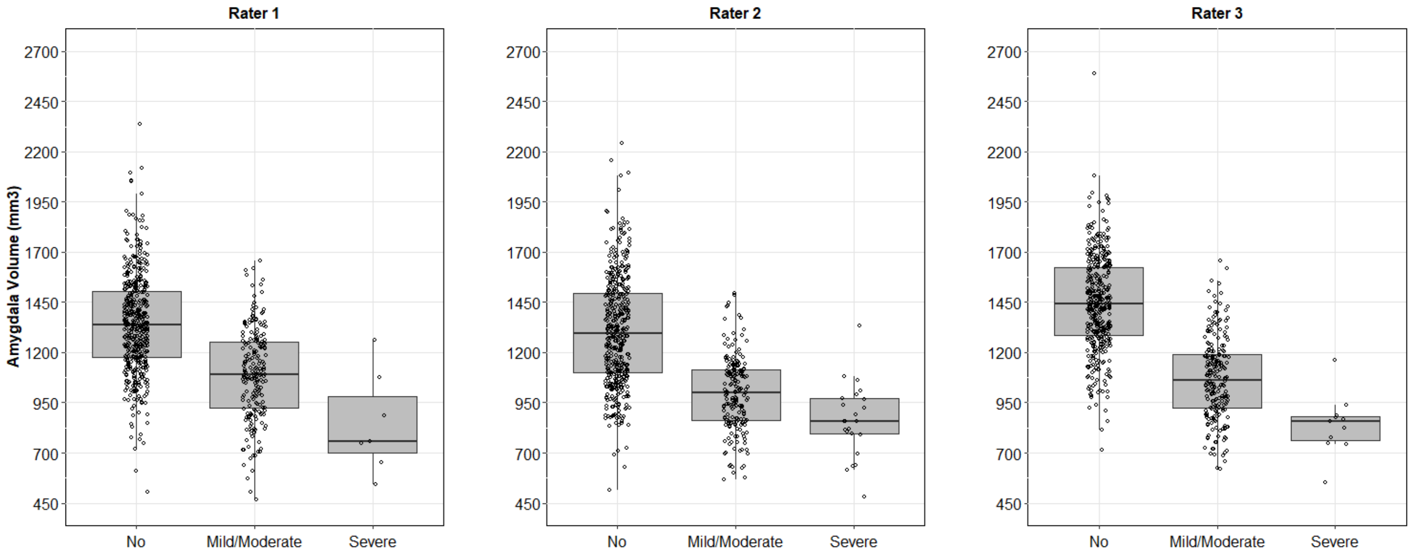

N	449	209	7		443	190	23		390	220	12
Mean ± SD	1342 ± 250	1088 ± 229	846 ± 250		1305 ± 271	992 ± 190	862 ± 182		1443 ± 240	1063 ± 213	795 ± 212
Median [1st -3rd quartile]	1337 [1178-1505]	1088 [923-1250]	759 [700-981]		1294 [1099-1497]	999 [860-1112]	858 [793-970]		1440 [1286-1623]	1057 [919-1189]	842 [745-879]

Abbreviations: SD Standard Deviation.

Supplementary Table 1. Medial temporal lobe atrophy by amygdalar atrophy scale (AAS) score. This table shows the non-redundancy of the AAS and MTA ratings. For each MTA value from 0 to 4, the number (column percentage), mean and standard deviation of the amygdalar volume scale scores (from no atrophy to severe) are shown.

		**Medial temporal lobe atrophy scale**				
**Amygdalar atrophy scale**		**0**	**1**	**2**	**3**	**4**
	**No atrophy**	295 (97%)<br>1483 ± 223	458 (88%)<br>1360 ± 237	149 (39.5%)<br>1186 ± 230	19 (11%)<br>1120 ± 164	1 (4%)<br>1388 ± NA
	**Mild/moderate atrophy**	8 (3%)<br>945 ± 201	60 (12%)<br>1154 ± 257	224 (60%)<br>1082 ± 206	140 (81%)<br>1024 ± 211	13 (52%)<br>959 ± 170
	**Severe atrophy**	0 (0%)<br>-	0 (0%)<br>-	2 (0.5%)<br>842 ± 23	13 (8%)<br>875 ± 245	11 (44%)<br>799 ± 109

Abbreviations: MTA Medial Temporal Atrophy

Supplementary Figure 2. This figure visually summarises Supplementary Table 1, showing that 56% of patients with MTA=4 have no atrophy or moderate atrophy of the amygdala and that MTA=3 has a non-negligible percentage of scans (8%) with severe atrophy of the amygdala. Whereas severe amygdala atrophy is extremely rare in scans with MTA of 2 or less.

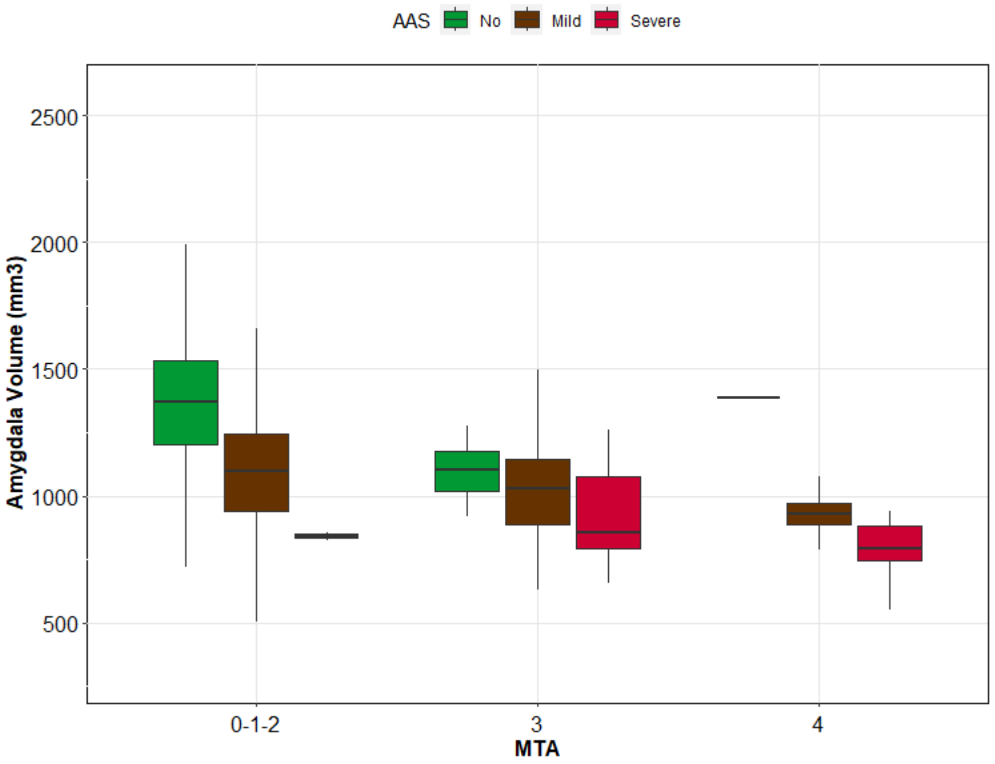

Abbreviations: MTA Medial Temporal Atrophy; AAS amygdalar atrophy scale

Supplementary Figure 3. Axial and coronal images of 4 subjects selected from the dataset presented in supplementary Table 1 and Figure 2 to exemplify the added value of AAS over MTA.

1. Normal ASS with high MTA value (MTA respectively =2 in Subject A and =3 in Subject B);
2. High AAS and low MTA: mild/moderate amygdala atrophy and MTA=1 (normal for age).

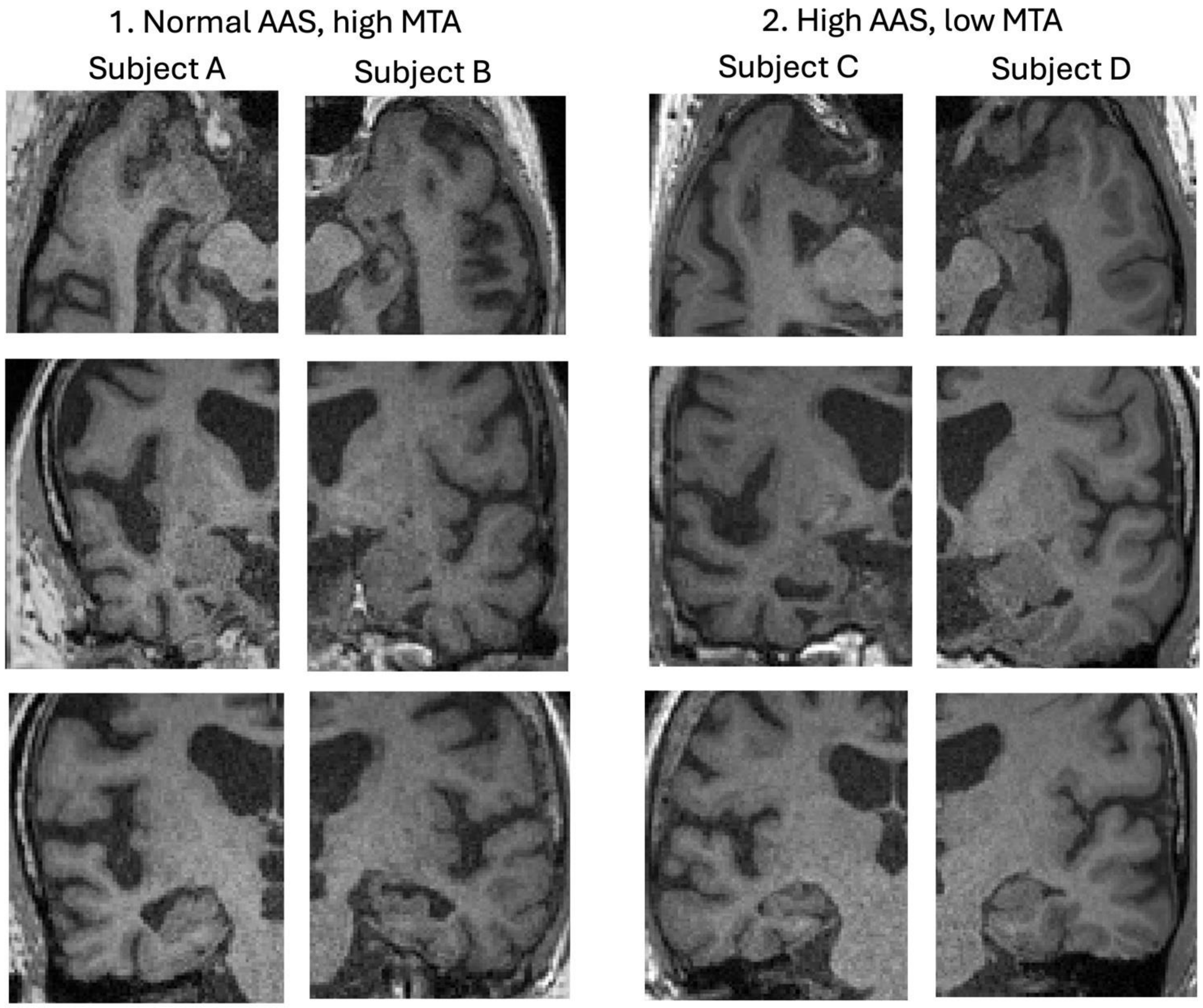

Abbreviations: MTA Medial Temporal Atrophy; AAS amygdalar atrophy scale
